# Detection of neutralizing antibodies against arboviruses from liver homogenates

**DOI:** 10.1371/journal.pntd.0012740

**Published:** 2024-12-13

**Authors:** Thaís Alkifeles Costa, Matheus Soares Arruda, Gabriela Fernanda Garcia-Oliveira, Erik Vinicius de Sousa Reis, Anna Catarina Dias Soares Guimarães, Gabriel Dias Moreira, Nidia Esther Colquehuanca Arias, Marina do Vale Beirão, Nikos Vasilakis, Kathryn A. Hanley, Betânia Paiva Drumond

**Affiliations:** 1 Laboratório de Vírus, Instituto de Ciências Biológicas, Universidade Federal de Minas Gerais, Belo Horizonte, MG, Brazil; 2 Department of Pathology, University of Texas Medical Branch, Galveston, Texas, United States of America; 3 Center for Vector-Borne and Zoonotic Diseases, The University of Texas Medical Branch, Galveston, Texas, United States of America; 4 Institute for Human Infection and Immunity, University of Texas Medical Branch, Galveston, Texas, United States of America; 5 Department of Biology, New Mexico State University, Las Cruces, New Mexico, United States of America; Medizinische Universitat Wien, AUSTRIA

## Abstract

Yellow fever virus (YFV) circulates in a sylvatic cycle between non-human primates (NHPs) and arboreal mosquitoes in Brazil. Passive monitoring of ill or deceased NHPs is a key component of the Brazilian yellow fever (YF) surveillance program. Samples from NHPs carcasses are usually suitable for molecular tests but not for serological assays. As an alternative to the conventional plaque reduction neutralization test (PRNT) based on sera, we tested the utility of liver homogenates from experimentally infected (YFV, Mayaro virus [MAYV], chikungunya virus [CHIKV], or mock) mice to quantify PRNTs. Although homogenates from mock-infected mice showed a low level of nonspecific virus neutralization against all three viruses, homogenates from YFV-, MAYV- and CHIKV-infected mice demonstrated significantly higher levels of virus neutralization compared to controls. Receiver operating characteristic (ROC) curves analyses were performed using the median neutralization values of three technical replicates for each infected group separately or collectively. Results showed scores ≥0.97 (95% CI ≥ 0.89–1.0) for the area under the curve at dilutions 1:20 to 1:80, suggesting that median virus neutralization values effectively differentiated infected mice from controls. Liver homogenates obtained from 25 NHP carcasses (collected during the 2017 YF outbreak in Brazil) were also tested using the adapted PRNT as well as rapid lateral flow tests to investigate anti-YFV IgM. Neutralization activity was detected in six NHP samples that were also positive by PCR and anti-YFV IgM tests and one sample that tested negative by PCR and IgM test. Our results demonstrate the feasibility of using liver homogenates as an alternative approach for serological investigation in viral epidemiologic surveillance.

## Author summary

Sylvatic yellow fever virus (YFV) is endemic in the Amazon Basin, where it is transmitted between non-human primates (NHP) by arboreal mosquitoes. Due to their high susceptibility to YFV, neotropical NHP are sentinels for YF. One of the arms of the yellow fever surveillance program in Brazil is passive monitoring of ill or deceased NHPs. Although samples from NHP carcasses are usually suitable for molecular tests, they are not suitable for serological assays limiting epidemiological investigations. Therefore, we aimed at evaluating the use of liver homogenates as alternative sample for the investigation of antibodies against arboviruses. Using liver homogenates from experimentally infected mice, we observed neutralizing activity against YFV, Mayaro virus (MAYV), and chikungunya virus (CHIKV), by plaque reduction neutralization assays (PRNT), indicating the presence of antibodies against these viruses. Next, we produced liver homogenates from NHP carcasses collected during YF outbreaks in Brazil and investigated the presence of antibodies against YFV using the adapted PRNT and rapid lateral flow tests for anti-YFV IgM. We detected neutralizing activity and IgM against YFV using liver homogenates from NHP carcasses previously confirmed to be infected with YFV. The results indicate that the use of liver homogenates can potentially contribute to serological surveillance endeavors, particularly when sera are not available.

## Introduction

Yellow fever virus (YFV) (*Flaviviridae*, *Orthoflavivirus*) is the causative agent of yellow fever (YF), a disease endemic in various tropical regions across Africa as well as Central and South America. Despite the existence of a highly effective vaccine, YF remains a significant global public health concern, as it is estimated that 80,000–200,000 YF cases occur annually in endemic regions, with a fatality rate ranging between 20% and 60% [1]. Central and South America ended urban transmission of YFV in the 1950s though massive immunization campaigns, however the sylvatic cycle persists and serves as the source of periodic spillover to humans and epidemics throughout the region [2,3].

In 2014, YFV reemerged in Midwest Brazil causing human cases and epizootics in the interface of the Brazilian Amazon, and Cerrado (Brazilian savannah) [4–7]. Subsequently, the virus spread to Southeastern Brazil, sparking a massive outbreak in Minas Gerais by December 2016, affecting thousands of humans and NHPs. Since then, YFV has spread into densely populated areas in Southeastern Brazil [2–5,7–9], indicating that this region has suitable ecological and climatic conditions for YFV maintenance during the epidemic and interepidemic seasons [5,10–12]. Between July 2014 and June 2023, 2,047 NHPs deaths; 2,289 human cases; and 780 human deaths (with a case fatality rate of 34%) caused by YFV were confirmed in the country [13–14].

Neotropical NHPs are very susceptible to YFV infection and are considered key sentinels for outbreaks in the Americas [2,3,10,15,16]. As reviewed by Silva and colleagues (2020) [3] YFV infection in NHPs is similar to humans, causing a viscerotropic disease, with viral replication in various organs (liver, kidneys, bone marrow, spleen, and lymph nodes). Viremia is typically short-lived, ranging from three to seven days post-infection, but virus replication is still observed in the liver after that [3]. One of the pillars of the Brazilian YF surveillance program is the investigation of NHPs epizootics. The early detection of YFV in NHPs can trigger control measures, such as human vaccination, thereby preventing outbreaks [3,16]. Epizootic surveillance primarily relies on passive monitoring of ill or deceased NHP of any species across Brazil. NHP carcasses constitute a primary source of biological samples for epizootic investigations, and laboratory tests are crucial for an accurate YF diagnosis [16].

The choice of laboratory tests to diagnose YF depends on the infection stage, the accessibility, and the quality of biological samples. For laboratory diagnostic purposes, viral isolation and molecular tests are recommended during the infection phase. The sensitivity of molecular tests hinges on sample quality and the amount of viral RNA [17]. Various factors, such as temperature, organ degradation, and duration of postmortem exposure to weather conditions prior to sample collection, may influence the preservation status of NHP carcasses, potentially compromising the quality and integrity of viral RNA and hindering detection [2,18–20]. After seroconversion, serological tests are preferred. IgM anti-YFV can be found in human serum samples six days post-exposure, and it may be detected up to four years in vaccinees [21–23]. As observed during the investigation of suspected YF human cases, the combined use of molecular and serological methods can enhance the sensitivity and specificity of the diagnosis [17]. However, the use of serological tests is frequently impractical due to difficulties or the impossibility of harvesting whole blood from carcasses [20,24]. On the other hand, samples from solid tissues are easily harvested from carcasses and previous studies have demonstrated the potential for detecting antibodies using pig muscle and using blood samples of human cadavers [25,26]. In this context, utilizing solid organs obtained from NHPs carcasses could serve as a valuable strategy for investigating the presence of antibodies against YFV.

An alternative strategy for serological investigation of viral infections in samples from NHP carcasses, coupled with molecular tests, could significantly contribute to our understanding of the ecology and dynamics of various zoonotic viruses. Since many NHPs establish specific territories [3], the detection of viral RNA or antibodies in local NHPs against specific viruses are good indicators of viral presence in a region. Besides YFV, NHPs act as significant arboviral reservoirs, creating the potential for spillover or spillback events for other orthoflaviviruses, such as Zika virus (ZIKV) [27] and alphaviruses like Mayaro (MAYV) and chikungunya (CHIKV) in Brazil [28–31]. In our study, we investigated the presence of neutralizing antibodies against YFV, MAYV, and CHIKV using liver homogenates from experimentally infected mice as a substitute for serum. The liver is the second largest organ in the body and has a role as a major blood reservoir, receiving 25% of cardiac output while constituting only 2.5% of body weight [32]. We selected liver as our target organ for screening because it: (*i*) has a higher likelihood of antibody recovery due to its significant blood supply; (*ii*) is easy to collect in larger quantities because of its size for preparing homogenates; and (*iii*) given the hepatic tropism of YFV, it is routinely collected from NHP carcasses, during YF surveillance programs. Subsequently, we applied the adapted protocol to investigate antibodies against YFV in liver samples from NHP carcasses collected during the YF epizootics in 2017 in Minas Gerais, Brazil.

## Material and methods

### Ethics statement

All the animal experiments received the approval of the Ethical Committee for Animal Experimentation of Universidade Federal de Minas Gerais (UFMG) (process no. 98/2017, approved in June ‐ 29/2017; 33/2021, approved in March ‐ 8/2021; 176/2021, approved in August ‐ 23/2021).

### Virus strains, mouse strains, infection, and non-human primate samples

The vaccine strain YFV-17DD (kindly provided by Dr. Pedro Augusto Alves, FIOCRUZ, Brazil), MAYV (BeAr20290, genotype L), and CHIKV (BHI1762H804917, genotype ECSA) (both kindly provided by Dr. Mauricio L. Nogueira, FAMERP, Brazil) were used for mouse infection and *in vitro* tests. ZIKV strain PE243 (kindly provided by Dr. Marli Tenório Cordeiro from FIOCRUZ-PE, Brazil) was used for *in vitro* tests.

Animal procedures were performed in mixed groups (males and females) of mice. For YFV experiments, IFNAR-/- mice (C57BL/6 background) aged 8 to 12 weeks were used. For MAYV and CHIKV experiments, C57BL/6 wild-type mice aged 3–4 weeks were used. Animals were housed in an animal care facility in individually ventilated cages at 23°C ± 2°C on a 12h/12h light/dark cycle with *ad libitum* access to water and food.

For each virus, mock (n = 6) and infected (n = 6) groups were utilized. One group infected with each virus was inoculated through the footpad route with 10 μL containing 1 x 10^3^ plaque-forming units (PFU) of YFV-17DD [33]; 1 x 10^5^ PFU of CHIKV [34]; or 1 x 10^5^ PFU of MAYV [35]. The mock-infected group was inoculated with the clarified supernatant of uninfected Vero cells [36]. Over 21 days, all animals survived and were observed for signs of infection. On day 21, mice were anesthetized with a solution of 100 mg/Kg of ketamine + 8–16 mg/Kg of xylazine per animal and were euthanized by cervical dislocation. Livers were harvested and stored at -80°C.

Liver samples collected from carcasses of free-living NHPs, during yellow fever outbreaks in 2017, in Minas Gerais, Brazil, were further utilized to obtain liver homogenates for serological testing. These samples were previously examined for YFV RNA by RT-qPCR [2].

### Sample processing

After euthanasia, liver samples were harvested from all mice (mock and infected groups), and separately processed to obtain a clarified liquid phase, referred to as a homogenate. Initially, liver samples were macerated utilizing four sterile beads in microtubes (Kasvi, USA) for 2 minutes in a bead beater with speed of 3,450 oscillations/min (Mini-Beadbeater-16, BioSpec Products, EUA), at a ratio of 50 mg of tissue to 200 μL of MEM without FBS. Subsequently, the samples were centrifuged at 16, 000 x g for 10 minutes, and the supernatant was transferred to new tubes, this clarification step was repeated five times. The homogenates were then incubated at 56°C without agitation (Thermomixer comfort, Eppendorf, Germany) for 30 minutes for heat inactivation of the immune complement system. During this step, a clotting-like reaction altered the appearance of the homogenate samples. In response, two additional rounds of sample clarification (16,000 x g for 10 minutes) were performed. The final clarified supernatants, referred to as liver homogenates, were stored at -20°C and utilized in cytotoxicity assays and for the viral neutralization tests.

Total RNA was extracted from approximately 30 mg of liver tissue from both infected and mock mice using the RNeasy Mini Kit (Qiagen, USA). Total RNA was then used to detect active infection by YFV [37], MAYV [38], or CHIKV [39] via RT-qPCR, as previously described [2,19].

Liver samples obtained from NHPs were also utilized to produce liver homogenates following the previously described method. The resulting liver homogenate was diluted 1:10 in MEM without FBS, filtered with a 0.22 μm cellulose acetate membrane syringe microfilter (Filtrilo, Brazil), and then used in PRNT. Additionally, undiluted liver homogenate from NHP was employed in rapid lateral flow tests for IgM or IgG against orthoflaviviruses such as YFV, dengue virus (DENV) and ZIKV.

### Cytotoxicity

To check whether liver homogenate could have a cytotoxic effect on Vero cells CCL-81, we performed 3-(4,5-dimethylthiazol-2-yl)-2,5-diphenyl-2H-tetrazolium bromide (MTT) reduction assay [40]. Vero cells (ATCC CCL-81) were cultivated in Eagle’s minimum essential medium (MEM) (Cultilab, Brazil) supplemented with 5% fetal bovine serum (FBS) (Cultilab, Brazil) and antibiotics (200 U/mL penicillin [Cellofarm, Brazil]; 40 μg/mL streptomycin, [Sigma-Aldrich, Germany]; and 2 μg/mL amphotericin B [Cultilab, Brazil]) and incubated in a humidified 5% CO_2_ atmosphere at 37°C. A total of 2.0 x 10^4^ Vero cells were seeded in each well of a 96-well plate and incubated for 24 h, at 37°C, and 5% CO_2_ atmosphere. Seventy microliters of liver homogenates were serially diluted (1:20 to 1:640) and added to Vero cells in 96-well plates in 200 μL of MEM 1% FBS, and incubated for two and five days, at 37°C, and 5% CO_2_ atmosphere. Afterward, the media was removed, and 28 μL of MTT solution (ThermoFisher Scientific, USA) in MEM (2 mg/mL) was added to each well. Cells were incubated at 37°C, and 5% CO_2_ atmosphere for 90 minutes protected from the light. Then, 130 μL of dimethyl sulfoxide solution (DMSO) was added to each well to solubilize formazan crystals. The plate, covered with aluminum foil, was agitated for 15 minutes. Finally, the absorbance at 540 nm was measured using a Multiskan GO Microplate spectrophotometer (Thermo Fisher Scientific, Waltham, Massachusetts, EUA), and the percentage decrease in cell viability was calculated. Simultaneously, under the same conditions, a crystal violet assay was performed to assess cell viability. In an identical plate, wells were washed with phosphate-buffered saline (PBS) three times, and 50 μL of 1% (w/v) crystal violet was added to each well and left to rest for 15 minutes. Plates were visually inspected regarding cell monolayer stability in the well. All conditions were tested in 12 well replicates.

### Adapted plaque reduction neutralization test (PRNT) with liver homogenate of experimentally infected mice

Liver homogenates from infected or mock mice, previously heat-inactivated and clarified, underwent triplicate testing by PRNT, in three technical replicates. Briefly, Vero CCL-81 cells (1.3 x 10^4^ cells per well) were seeded into 12-well plates and incubated at 37°C with a 5% CO_2_ atmosphere for 24 hours. Each virus was diluted using MEM 1% FBS, to achieve approximately 100–150 PFU per well. Liver homogenate samples (80 μL) were two-fold serially diluted (1:20 to 1:160) in MEM 1% FBS 1% HEPES buffer (4-[2-hydroxyethyl]-1-piperazineethanesulfonic acid).

Equal volumes of virus and diluted liver homogenate (1:10 to 1:80) were mixed, resulting in final dilutions of 1:20 to 1:160 of liver homogenates, and incubated at 37°C for 1 hour. Subsequently, 180 μL of the virus/liver homogenate mixture (1:20 to 1:160) was inoculated per well (in triplicate) onto Vero CCL-81 cells. Plates were incubated at 37°C for 1 hour at 5% CO_2_ atmosphere, with gentle rocking every 15 minutes. After incubation, the inoculum was removed, and each well was covered with 1 mL of 199 medium supplemented with 2% FBS and carboxymethyl cellulose (1% for YFV assay and 1.5% for CHIKV and MAYV assays) and plates were incubated at 37°C, 5% CO_2_ atmosphere. For YFV assay, plates were incubated for five days, while for CHIKV and MAYV assays, plates were incubated for two days, until the emergence of viral lysis plaques. Following incubation, plates were fixed with 3.7% formaldehyde solution for 1 hour and stained with 1% crystal violet solution for 30 minutes. To investigate cross-neutralization, liver homogenates of YFV-infected mice (individuals 2, 3, and 4) were used in PRNT using ZIKV PE243, under the same experimental conditions described above and four days of incubation until the emergence of viral lysis plaques.

The serum used as positive controls were obtained from a biobank of samples from previously experimentally infected mice, as described above. Unfortunately, there were insufficient volumes of sera from the experimentally YFV-, MAYV-, CHIKV- or mock infected mice groups to be tested in parallel to liver homogenates. To assess whether liver homogenate could interfere with the neutralization activity, a second positive control, consisting of a 1:1 mixture of positive sera and liver homogenate from uninfected mice, was used. Liver homogenate from uninfected mice served as negative control. All samples and controls were tested in triplicate. Each plate included a well designated for cell control (no virus inoculation) and two wells designated for virus control.

Assay validity criteria included (*i*) the absence of PFU in the cell control well; (*ii*) neutralization by positive control sera, and no neutralization by negative control sera; (*iii*) the observed number of PFU for each triplicate (per each dilution of each mock or infected sample tested) in an experiment were within a 3-fold difference of the median PFU count [41,42] and; (iv) the observation of 70 to 180 PFU in virus control wells. The median percentage of viral neutralization was estimated for each experiment comparing the median number of PFUs in virus wells to those in wells where the virus was incubated with liver homogenates, positive and negative controls. Three independent assays were conducted, with all PRNT assays completed by five researchers following the same standard assay procedures.

### Serological tests using liver homogenates of non-human primates

Filtered liver homogenates from NHPs were subjected to PRNT using YFV (dilutions 1:20 to 1:640), following the previously described method. NHP liver homogenates which presented neutralizing activity against YFV were also tested in PRNT using ZIKV (dilutions 1:20 to 1:160) to investigate cross-neutralization, as described above. Undiluted liver homogenates were also tested by rapid lateral flow assays to investigate the presence of IgM anti-YFV, IgG/IgM anti-Dengue virus (DENV), and IgG/IgM anti-ZIKV virus (Febre amarela IgM EcoTest, Dengue IgG/IgM EcoTeste, and Zika IgG/IgM EcoTeste ‐ EcoDiagnóstica, Brazil) following the manufacturer’s instructions. Briefly, 10 μL of liver homogenate were deposited in the specimen well of the cassettes. Subsequently, three drops of the buffer provided by the manufacturer were added, and a 15-minute timer was set for result revelation.

### Data analyses

Statistical analyses were performed using GraphPrism version 8.0.2 for Windows (GraphPad Software, USA) or RStudio 4.3.2 [43,44] with lme4 [45]. For cytotoxicity analysis, linear regression was applied, with results with r^2^ > 0.9. Comparisons were made between the mock and infected groups for each homogenate dilution, and the differences were considered statistically significant when p ≤ 0.05. Generalized linear mixed models (GLMM) were used to evaluate the neutralization response to the infection status of each animal, categorized as infected or mock groups. Individuals (animals) were treated as the random variable, while the explanatory variables included group (infected or mock), dilution (1:20–1:160), and the interaction group/dilution. The response variable was virus neutralization. In different GLMM analyses, we used median neutralization values for both infected and mock groups (values per animal, per dilution, obtained from three independent assays (S1 Table). All models were submitted to residual analysis to evaluate adequacy of error distribution [46]. Minimum adequate models were generated by stepwise omission of non-significant terms. Paired analyses (virus neutralization per group [mock or infected mice] per dilution) were conducted using GLMM with the group as the response variable and the animal as the random variable.

GLMM were also used to assess how neutralization of mock or infected animals responded to the virus used in each assay (YFV, MAYV and CHIKV), designated by virus, by dilution (1:20 to 1:160), and by the interaction virus/dilution. Analyses were carried out considering mock and infected groups separately. Individuals were treated as the random variable, while the explanatory variables included virus (YFV, MAYV or CHIKV), dilution, and the interaction virus/dilution. The response variable was median virus neutralization (values per animal, per dilution), obtained from three independent assays (S1 Table). Post-hoc analyses for pairwise comparisons were performed, with Tukey correction, using the glht function in Multcomp package [47] and the differences were considered statistically significant when p ≤ 0.05.

Neutralization percentage data were subjected to Receiver operating characteristic (ROC) curve analyses performed using GraphPad Prism version 8.0.2 for Windows (GraphPad Software, USA) using the Wilson/Brown method [48,49]. For these analyses, median values (S1 Table) were compared by dilution for YFV, MAYV, and CHIKV separately. Additionally, we performed the same analysis using median neutralization values for the three viruses combined (S1 Table), also grouped by dilution. Moreover, we compared the lowest median neutralization values obtained from infected animal samples with the highest neutralization values obtained from mock-infected samples for each dilution, to investigate the occurrence of overlapping neutralization amongst both groups (S1 Table).

## Results and discussion

### Neutralizing activity of liver homogenates of experimentally infected mice against YFV, MAYV, and CHIKV

To test whether virus-neutralizing antibodies could be detected from the livers of animal carcasses, we employed liver homogenates obtained from mice experimentally infected with YFV, MAYV, CHIKV, or subjected to mock infection for performing PRNTs. The choice of liver was motivated by the fact that it is an easily accessible sample, with a significant amount of blood [32], coupled with the advantage of using a biological sample that is routinely collected during YF surveillance in Brazil [16]. We infected mice with non-lethal doses of YFV, MAYV, and CHIKV and evaluated them for infection signs up to 21 dpi. All YFV-infected mice exhibited mild signs of infection, including ruffled fur and hunched posture, up to 10 days post-infection (dpi). In contrast, CHIKV- or MAYV-infected mice only presented paw swelling observed between the second and third dpi. Mock-infected animals presented no reaction to mock components. For all viruses, all animals survived until 21 dpi, and at this date, we euthanized animals and harvested the liver without venipuncture. At 21 dpi, all liver samples were negative for the presence of YFV, MAYV, or CHIKV RNA by RT-qPCR.

Cytotoxicity of liver homogenates for Vero cells was then assessed. Liver homogenates of C57BL/6 (n = 3) and IFNAR -/- (n = 4) animals were incubated in Vero CCL-81 cells for two and five days, respectively, and cytotoxicity was evaluated by an MTT assay. At two days of incubation (S1 Fig), the mean cell viability was 80.1% at the 1:20 dilution, and the viability was > 80% from dilution 1:40 on. At five days of incubation (S1 Fig), at the 1:20 dilution, the mean cell viability was 75.97%, and above that (1:40 and beyond) the viability was > 80% (S1 Fig). In parallel, cells treated under the same conditions were fixed and stained with crystal violet 1%. The results indicated that cell monolayer integrity was preserved supporting that liver homogenate was not cytotoxic.

Next, the adapted PRNT was performed, with serum being replaced by the clarified liver homogenate. Liver homogenates from both mock and YFV-infected animals were evaluated in neutralization assays against YFV. From dilution 1:20 to 1:160, the median virus neutralization decreased in the YFV-infected (63.5 to 7.4%) and in the mock group (24.0 to 0.0%), across three independent assays (Table 1). When liver homogenates of mock or MAYV-infected animals were tested against MAYV in adapted PRNT, decreasing median neutralization values were also observed for both MAYV-infected (63.5 to 9.2%) and mock groups (22.9 to 1.5%), from dilution 1:20 to 1:160 (Table 1). Likewise, liver homogenates of mock- or CHIKV-infected animals were evaluated for their response to CHIVK using the adapted PRNT. From dilution 1:20 to 1:160, the median virus neutralization decreased in CHIKV-infected group (59.2 to 11.7%) while median neutralization was equal to 0% in all dilutions for the mock group (Table 1). In addition, the neutralization of YFV, MAYV, or CHIKV was not prevented by the liver homogenate, as demonstrated by the median neutralization values observed for the positive control 2 (1:1 mixture of liver homogenate from uninfected mice and sera from infected mice) compared to positive control 1 (constituted of sera from infected mice) (Table 1).

**Fig 1:**
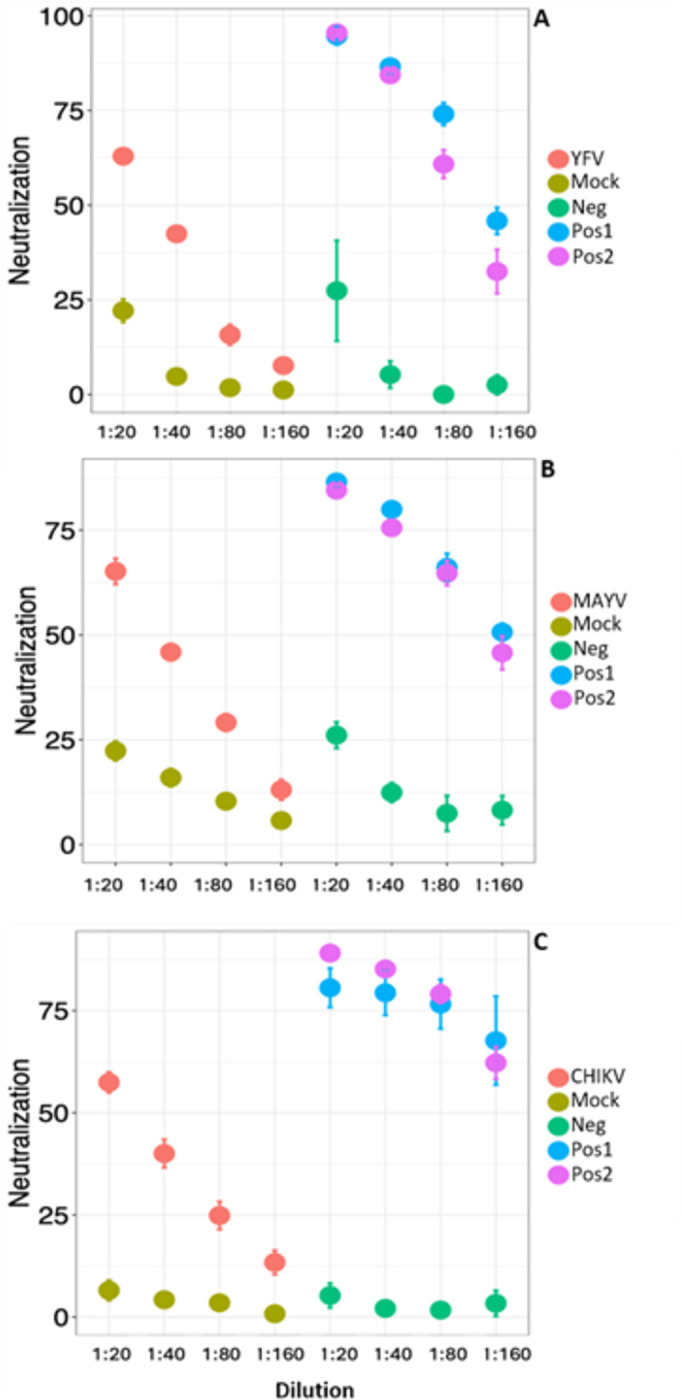
Average virus neutralization values of YFV (A), MAYV (B), CHIKV (C) per dilution, using liver homogenates from experimentally infected mice in adapted plaque reduction neutralization tests. A, B and C respectively display average neutralization values (in percentage) obtained from the experiments carried out with samples from YFV-, MAYV- and CHIKV-infected groups, and their respective mock-groups. Bars represent standard deviation. YFV-, MAYV-, and CHIKV- infected groups consisted of six mice each as well the respective mock-infected groups. The negative control is liver homogenate obtained from uninfected animals (neg), the positive control is sera from experimentally infected animal (pos 1), and the second positive control is composed of a mixture (1:1) of liver homogenate of uninfected animal and positive serum (pos2).

Generalized linear mixed model analyses revealed a significant interaction between group and dilution (p < 0.001, Table 2 and Fig 1). The results indicated that differences in neutralization between infected and mock groups were substantial at low serum dilutions but diminished as dilution increased. This pattern could be explained by the dilution of specific neutralizing antibodies in liver homogenates of infected mice, in contrast to their absence in the mock group (Table 2 and Fig 1). Similarly, pairwise analyses at each dilution demonstrated that the difference in average neutralization between the YFV-, MAYV-, and CHIKV-infected groups and their respective mock groups decreased up to dilution 1:80 (p <0.01, see the bold numbers in Table 3). This difference between infected and mock groups could be attributed to the presence of specific neutralizing antibodies in the liver homogenates of infected mice in contrast to their absence in mock mice.

**Table 1:** Median virus neutralization values (%) of YFV, MAYV, CHIKV, per dilution and group, using liver homogenates from experimentally infected mice in adapted plaque reduction neutralization tests.

Virus	Group	Median percentage virus neutralization (range)			
		Dilution 1:20	Dilution 1:40	Dilution 1:80	Dilution 1:160
YFV	Infected	63.5 (45.7–72.2)	42.9 (24.3–53.2)	19.1 (0.0–31.6)	7.4 (0–21.3)
	Mock	24.0 (0.0–38.4)	2.5 (0.0–20.8)	0 (0.0–25.6)	0.0 (0.0–13.6)
	Pos 1	96.5 (85.6–99.1)	84.8 (82.0–92.9)	77.6 (63.52–79.5)	50.0 (32.1–50.7)
	Pos 2	95.7 (91.8–97.5)	85.11(79.6–89.9)	56.9 (53.7–73.4)	33.3 (14.9–50.9)
	Neg	29.2 (0.8–50.5)	0.0 (0.0–17.9)	0.0 (0.0–0.0)	0.0 (0.0–12.8)
MAYV	Infected	63.5 (39.5 84.8)	46.6 (26.1–57.5)	32.4 (16.2–43.5)	9.2 (0.0–28.5)
	Mock	22.9 (7.6–37.2)	16.6 (0–32.6)	8.0 (0–24.5)	1.5 (0.0–23.0)
	Pos 1	85.4 (84.0–91.5)	79.9 (78.2–81.7)	66.5 (52.1–76.8)	49.3 (45.8–58.7)
	Pos 2	85.9 (78.5–88.1)	75.3 (73.1–79.0)	65.4 (54.4–74.4)	42.3 (36.7–63.8)
	Neg	25.9 (14.9–37.7)	14.0 (1.7–17.0)	2.84 (0.0–25.7)	7.1 (0.0–22.3)
CHIKV	Infected	59.2 (38.6–73.6)	38.5 (11.9–68.2)	22.7 (3.6–51.8)	11.7 (0.0–41.0)
	Mock	0.0 (0.0–32.5)	0.0 (0.0–19.6)	0.0 (0.0–19.1)	0.0 (0.0–10.1)
	Pos 1	85.4 (58.0–89.9)	81.8 (58.0–94.9)	75.2 (60.7–94.2)	69.2 (34.3–94.1)
	Pos 2	89.8 (84.5–90.8)	84.9 (77.5–91.0)	79.9 (70.8–85.9)	61.3 (51.4–78.2)
	Neg	1.32 (0.0–16.7)	0.27 (0.0–6.4)	0.0 (0.0–8.4)	0.0 (0.0–19.1)

YFV: Yellow fever virus, MAYV: Mayaro virus, CHIKV: Chikungunya virus. Median virus neutralization values using liver homogenates in adapted plaque reduction neutralization assays are shown in percentages (results from three independent assays). YFV-, MAYV-, and CHIKV- infected groups consisted of six mice as well as the respective mock-infected groups. The negative control is liver homogenate obtained from uninfected animals (Neg), one positive control is sera from experimentally infected animals (Pos 1), and the second positive control is composed of a mixture (1:1) of liver homogenate of uninfected animal and positive serum (Pos2).

**Table 2:** Results of generalized linear mixed models’ analysis of the response of average virus neutralization to groups infected and mock, dilution and group/dilution in adapted plaque reduction neutralization tests using liver homogenates of experimentally infected mice.

Response variable	Explanatory variables	F	P
YFV neutralization (%)	group (YFV infected/mock)	309.13	< 0.001
	dilution	154.13	< 0.001
	group/dilution	37.45	< 0.001
MAYV neutralization (%)	group (MAYV infected/mock)	204.41	< 0.001
	dilution	72.97	< 0.001
	group/dilution	19.31	< 0.001
CHIKV neutralization (%)	group (CHIKV infected/mock)	283.58	< 0.001
	dilution	35.66	< 0.001
	group/dilution	22.07	< 0.001
YFV + MAYV + CHIKV neutralization (%)	group (infected/mock)	590.48	< 0.001
	dilution	168.85	< 0.001
	group/dilution	55.23	< 0.001

YFV: Yellow fever virus, MAYV: Mayaro virus, CHIKV: Chikungunya virus. YFV-, MAYV-, and CHIKV- infected groups consisted of six mice each as well the respective mock-infected groups.

**Table 3:** Pairwise comparison of average virus neutralization per group/dilution in adapted plaque reduction neutralization tests using liver homogenates of experimentally infected mice.

**virus group/dilution**		**Mock (YFV)**				**YFV-infected**		
		1:20	1:40	1:80	1:160	1:40	1:80	1:160
**YFV-Infected** **Mock** **(YFV)**	1:20	<0.01*	<0.01*	<0.01*	<0.01*	<0.01*	<0.01*	<0.01*
	1:40	<0.01*	<0.01*	<0.01*	<0.01*		<0.01*	<0.01*
	1:80	<0.01*	<0.01*	<0.01*	<0.01*			0.067
	1:160	<0.01*	0.970	0.397	**0.272**			
	1:40	<0.01*						
	1:80	<0.01*	0.964					
	1:160	<0.01*	0.908	0.888				
**virus group/dilution**		**Mock (MAYV)**				**MAYV-infected**		
		1:20	1:40	1:80	1:160	1:40	1:80	1:160
**MAYV ‐ Infected** **Mock** **(MAYV)**	1:20	<0.01*	<0.01*	<0.01*	<0.01*	<0.01*	<0.01*	<0.01*
	1:40	<0.01*	<0.01*	<0.01*	<0.01*		<0.01*	<0.01*
	1:80	0.511	<0.01*	<0.01*	<0.01*			<0.01*
	1:160	0.128	0.991	0.994	**0.402**			
	1:40	0.589						
	1:80	0.012*	0.734					
	1:160	0.01*	0.062	0.888				
**virus group/dilution**		**Mock (CHIKV)**				**CHIKV-infected**		
		1:20	1:40	1:80	1:160	1:40	1:80	1:160
**CHIKV -Infected** **Mock (CHIKV)**	1:20	<0.001*	<0.001*	<0.001*	<0.001*	<0.001*	<0.001*	<0.001*
	1:40	<0.001*	<0.001*	<0.001*	<0.001*		<0.001*	<0.001*
	1:80	<0.001*	<0.001*	<0.001*	<0.001*			0.029*
	1:160	0.543	0.175	0.106	0.011*			
	1:40	0.998						
	1:80	0.990	1.000					
	1:160	0.754	0.980	0.996				
**virus group/dilution**		**Mock (all viruses)**				**all viruses-infected**		
		1:20	1:40	1:80	1:160	1:40	1:80	1:160
**all viruses-infected** **mock-** **(all viruses)**	1:20	<0.001*	<0.001*	<0.001*	<0.001*	<0.001*	<0.001*	<0.001*
	1:40	<0.001*	<0.001*	<0.001*	<0.001*		<0.001*	<0.001*
	1:80	0.070	<0.001*	<0.001*	<0.001*			<0.001*
	1:160	0.184	0.871	0.086	0.001*			
	1:40	0.003*						
	1:80	<0.001*	0.832					
	1:160	<0.001*	0.138	0.931				

YFV: Yellow fever virus, MAYV: Mayaro virus, CHIKV: Chikungunya virus. Post-hoc analyses of generalized linear mixed models, with Tukey correction. The asterisks indicate statistical significance, and the bold numbers indicate the comparison between groups in the respective dilution.

The average neutralization differed between serial dilutions (p< 0.029) (e.g. 1:20 vs 1:40, 1:20 vs 1:80 and so on) within each infected group (YFV, MAYV, CHIKV, and all viruses combined) (Table 3) with a single exception (YFV-infected group, dilution 1:80 vs. 1:160, Table 3). The variations in viral neutralization observed among serial dilutions of the infected group may be linked to the presence of specific neutralizing antibodies in the liver homogenates of infected mice, which diminish as the dilution increases. On the other hand, differences in the average median neutralization values for mock groups were only observed when the lowest dilution [1,20] was compared to higher dilutions within each mock group (Table 3). These results indicate that in the dilution 1:20, a higher nonspecific neutralization could be observed, but as the dilution factor increased from dilution 1:40 on, no significant effect on viral neutralization was observed and the reduction on PFUs observed for the mock group could be related to nonspecific neutralization.

To assess whether the average neutralization values could differ depending on the virus used in each assay, we separated mock from infected groups and performed GLMM analyses considering virus, dilution, and the interaction of virus and dilution. When YFV-, MAYV- and CHIKV-infected groups were compared to each other, the type of virus (p = 0.003) and dilution (p< 0.001) significantly affected the average neutralization (S2 Table and S2 Fig). When mock groups were compared to each other, a significant interaction between virus and dilution (p< 0.001) was observed (S2 Table and S3 Fig), indicating that as dilution increased the difference among types of viruses waned. This could be explained by the dilution of components of liver homogenate leading to nonspecific neutralization of all viruses investigated. In addition, post-hoc pairwise comparisons demonstrated that non-specific neutralization varied among viruses in different dilutions (S3 Fig). The results altogether showed that when using liver homogenates, we can observe virus neutralization for all viruses investigated here and that nonspecific neutralization may vary depending on the virus investigated and the dilution.

The data revealed a baseline virus neutralization in both mock and infected groups, potentially attributable to the composition of the liver homogenate. Even after multiple rounds of clarification, it is plausible that some residual cellular or tissue debris persists as particulate organic matter in the liver homogenate. This particulate organic matter might interfere with the viral infection process by adsorbing to viral particles or the cell monolayer, leading to nonspecific virus neutralization. Despite the nonspecific virus neutralization observed in mock groups, infected mice exhibited higher virus neutralization compared to mock mice, which we attribute to the presence of neutralizing antibodies enacting specific virus neutralization. Moreover, when liver homogenates of YFV-infected mice were tested against ZIKV, median neutralization values of 7.6% to 2.2% were observed from dilution 1:20 to 1:160, revealing no cross neutralization of ZIKV by anti-YFV antibodies within the liver homogenates of YFV-infected mice (S3 Table).

ROC curve analyses were conducted utilizing the median neutralization values from three independent assays for each infected group separately (YFV, MAYV, or CHIKV) and collectively (YFV+MAYV+CHIKV), alongside their corresponding mock groups. AUC scores ≥0.97 (95%CI ≥0.89–1.0, p<0.0065) indicated that the median virus neutralization values effectively differentiated YFV-, MAYV- or CHIKV-infected groups from their respective mock groups at dilutions 1:20 to 1:80 (Table 4). The ROC curve analyzes in agreement with GLMM results, confirmed that median virus neutralization values successfully discriminated between infected and mock groups across dilutions 1:20 to 1:80 for all viruses.

**Table 4:** Receiver operating characteristic (ROC) curve analyses for median neutralization values of YFV-, MAYV-, CHIKV-infected groups compared to their respective mock groups.

Virus (sample size)	Dil	Median neutralization values infected x mock		Cutoff	Se (95% CI)	Sp (95% CI)
		AUC (95% CI)	p value			
YFV (n = 12)	1:20	1.0 (1.0–1.0)	0.0039*	57.96	0.83 (0.44–0.99)	1.0 (0.61–1.0)
	1:40	1.0 (1.0–1.0)	0.0039*	40.85	0.83 (0.44–0.99)	1.0 (0.61–1.0)
	1:80	1.0 (1.0–1.0)	0.0039*	17.39	0.83 (0.44–0.99)	1.0 (0.61–1.0)
	1:160	0.92 (0.73–1.0)	0.0163*			
MAYV (n = 12)	1:20	1.0 (1.0–1.0)	0.0039*	56.59	0.83 (0.44–0.99)	1.0 (0.61–1.0)
	1:40	1.0 (1.0–1.0)	0.0039*	39.91	0.83 (0.44–0.99)	1.0 (0.61–1.0)
	1:80	0.97 (0.89–1.0)	0.0065*	24.59	0.83 (0.44–0.99)	1.0 (0.61–1.0)
	1:160	0.72 (0.42–1)	0.20			
CHIKV (n = 12)	1:20	1.0 (1.0–1.0)	0.0039*	46.04	0.83 (0.44–0.99)	1.0 (0.61–1.0)
	1:40	1.0 (1.0–1.0)	0.0039*	30.13	0.83 (0.44–0.99)	1.0 (0.61–1.0)
	1:80	1.0 (1.0–1.0)	0.0039*	11.39	0.83 (0.44–0.99)	1.0 (0.61–1.0)
	1:160	0.92 (0.73–1.0)	0.0163*			
All viruses (n = 36)	1:20	1.0 (1.0–1.0)	<0.0001*	55.04	0.83 (0.61–0.94)	1.0 (0.82–1.0)
	1:40	1.0 (1.0–1.0)	<0.0001*	36.36	0.83 (0.61–0.94)	1.0 (0.82–1.0)
	1:80	0.97 (0.92–1.0)	<0.0001*	17.53	0.83 (0.61–0.94)	0.94 (0.74–0.99)
	1:160	0.84 (0.70–0.98)	0.0004*			

Median virus neutralization values using liver homogenates in adapted plaque reduction neutralization assays were used for ROC analyses. Samples were grouped (n = 12; 6 infected + 6 mock) and compared by dilution. Analyses were made for each virus separately and also considering all viruses combined (all) (n = 36). YFV: Yellow fever virus, MAYV: Mayaro virus, CHIKV: Chikungunya virus. AUC: area under the curve. Se: sensitivity, Sp: specificity, dil: dilution. Analyses were carried out with GraphPad Prism 8.0.2. The asterisks indicate statistical significance.

Using a more conservative approach, we ran ROC curves comparing the lowest median neutralization values from the infected groups and the highest median neutralization values from the mock groups. The analyses showed that the YFV-, MAYV- and CHIKV-infected groups were clearly distinguished from the mock groups at dilutions 1:20 and 1:40 (AUC ≥0.92, 95%CI 0.74–1.0, p≤0.0163) (S4 Table). This aligns with the GLMM results, as the discrepancy between the neutralization values of infected and mock groups is more pronounced at lower dilutions and diminishes with increasing factors, suggesting greater discriminatory power between infected and uninfected groups at lower dilutions.

The PRNT titer should be calculated based on a 50% or greater reduction in plaque counts to be considered positive. Regrettably, we did not have adequate volumes of sera from experimentally infected mice to run neutralization assays concurrently and assess the level of neutralizing antibodies in sera in comparison to liver homogenates. The decision to refrain from blood harvesting aimed to replicate the natural condition of carcasses, preserving the blood quantity in the liver of experimental animals, and maximizing the chances of detecting neutralizing antibodies in liver homogenates. Given the presence of nonspecific neutralization in all samples, cutoffs were initially estimated to achieve 83% sensitivity and 100% specificity, enhancing the likelihood of identifying true positives and preventing false positives. Targeting 83% of sensitivity and 100% of specificity, for dilution 1:20, cutoffs of 57.96, 56.59, 46.04, and 55.04% neutralization were estimated for YFV, MAYV, CHIKV, and all viruses combined, respectively (Table 4). For dilution 1:40, cutoffs of 40.85, 39.91, 30.13, and 36.36% were estimated for YFV, MAYV, CHIKV, and all viruses combined, respectively (Table 4). We recommend considering a more conservative approach, incorporating a cutoff of 60% neutralization at dilution 1:20 and 40% at dilution 1:40 (sensibility of 0.67 (95%CI 0.44–0.84) and specificity of 1.0 (95%CI 0.82–1.0), while also observing the proportional reduction in neutralization as the dilution factor increases.

### Investigation of YFV neutralization using liver homogenates from carcasses of free-living neotropical non-human primates

Having established the feasibility of detecting specific virus neutralization activity in liver homogenates from experimentally infected animals, we opted to test liver homogenates obtained from carcasses of free-living NHP collected in rural areas, during the YF outbreak in Minas Gerais, Brazil, in 2017. Frozen liver samples were used to prepare the liver homogenates. Liver homogenates were clarified as described, and an additional step of filtration was added to enhance the clarification process and prevent bacterial contamination from the carcasses. We used 25 samples from NHP carcasses that had undergone prior screening for YFV by RT-qPCR [2], including 8 samples positive for YFV infection confirmed by RT-qPCR and 17 negative ones (Table 5). The carcasses belonged to animals of different genera, including *Alouatta* sp., *Callicebus* sp., and *Callithrix* sp. that were considered in a good (n = 14), intermediate (n = 10), or bad (n = 1) preservation status, based on previously established criteria [2].

Twenty-five samples, previously examined using RT-qPCR for the YFV genome, underwent further testing using YFV-PRNT, with analysis conducted based on predefined cutoffs and the observing gradual reduction in neutralization proportionally to the increase in dilution factor. Seven samples were positive by the adapted PRNT (Table 5), as they showed neutralization values ranging from 71.2% to 99.2% at dilution 1:20 and from 47.7% to 94.3% at dilution 1:40 (S5 Table). The positive samples in YFV-PRNT belonged to specimens of *Callithrix sp*., *Callicebus sp*., and *Alouatta sp*. Also, it was possible to detect antibodies in carcasses that were considered in a good or intermediate status of conservation (Table 5). Six of these samples that were YFV positive were further assayed for cross-neutralization using ZIKV in the adapted in PRNT. None of the samples were considered positive based on the established cutoffs (S5 Table), indicating the lack of cross-neutralization of ZIKV by antibodies in liver homogenate of NHP carcasses (Table 5), consistent with our findings from mice.

Following the PRNT, undiluted liver homogenates from the 25 samples were subjected to lateral flow tests for IgM against YFV, and IgG and IgM against DENV and ZIKV. All samples yielded positive results in the control test (control bands were visible 2 minutes after the test started). Six out of seven positive samples in PRNT and PCR were positive for IgM anti-YFV. Two of these samples were positive for IgG anti DENV and anti ZIKV, indicating a cross reaction with YFV (Table 5). Eighteen samples were also considered negative in the YFV-PRNT (Table 5), according to the established cutoffs (S5 Table). None of these samples were positive in rapid lateral flow tests for IgM or IgG against the orthoflaviviruses tested here (Table 5).

**Table 5:** Investigation of antibodies against orthoflaviviruses using liver homogenates from non-human primate carcasses.

Sample/carcass		RTqPCR		PRNT		Rapid lateral flow tests		
Id	Taxa (status)		Cq	YFV	ZIKV	IgM YFV	IgG/IgM DENV	IgG/IgM ZIKV
49	*A*. *guariba* (G)	P	10.5	P (1:80)	N	P	P/N	P/N
29	*C*. *personatus* (G)	P	8.5	P (1:40)	N	P	N/N	N/N
205	*Callithrix sp* (G)	N	-	P (1:40)	N	N	N/N	N/N
26	*C*. *penicillata* (I)	P	14.0	P (1:40)	N	P	N/N	N/N
174	*C*. *nigrifrons* (G)	P	22.5	P (1:40)	N	P	N/N	N/N
10	*C*. *nigrifrons* (G)	P	17.5	P (1:40)	N	P	P/N	P/N
56	*C*. *nigrifrons* (G)	P	8.0	P (1:40)	N	P	N/N	N/N
36	*Callithrix sp* (I)	N	-	N	-	N	N/N	N/N
341	*C*. *geoffroyi* (G)	N	-	N	-	N	N/N	N/N
14	*C*. *penicillata* (I)	N	-	N	-	N	N/N	N/N
220	*Callithrix sp* (I)	N	-	N	-	N	N/N	N/N
62	*C*. *penicillata* (I)	P	35.3	N	-	N	N/N	N/N
50	*A*. *guariba* (G)	N	-	N	-	N	N/N	N/N
63	*C*. *penicillata* (I)	N	-	N	-	N	N/N	N/N
287	*C*. *geoffroyi* (I)	N	-	N	-	N	N/N	N/N
11	*C*. *penicillata* (G)	N	-	N	-	N	N/N	N/N
165	*C*. *nigrifrons* (I)	N	-	N	-	N	N/N	N/N
338	*C*. *nigrifrons* (G)	N	-	N	-	N	N/N	N/N
53	*C*. *penicillata* (G)	N	-	N	-	N	N/N	N/N
251	*C*. *penicillata* (I)	N	-	N	-	N	N/N	N/N
233	*C*. *penicillata* (G)	N	-	N	-	N	N/N	N/N
255	*C*. *geoffroyi* (G)	N	-	N	-	N	N/N	N/N
77	*C*. *nigrifrons* (B)	P	34.2	N	-	N	N/N	N/N
61	*C*. *penicillata* (I)	N	-	N	-	N	N/N	N/N
212	*C*. *penicillata* (G)	N	-	N	-	N	N/N	N/N

RT-qPCR: reverse transcription polymerase chain reaction. PRNT: plaque reduction neutralization test. Results of PRNT are shown as negative (N) or positive (P) and the highest dilution which presented neutralization equal of above 60% is indicated in brackets. ID: sample identification. P: positive; N: negative and -: not available. YFV: Yellow fever virus. ZIKV: Zika virus. *A*. *guariba*: *Alouatta guariba; C*. *penicillata*: *Callithrix penicillata; C*. *geoffroyi*: *Callithrix geoffroyi; C*. *personatus*: *Callicebus personatus; C*. *nigrifrons*: *Callicebus nigrifrons*. Carcasses were previously classified regarding the preservation status as good (G), intermediate (I), or bad (B) (see Sacchetto et al, 2020[2]. Results of previous RT-qPCR performed on NHP liver/or brain samples with information on the cycle quantification value (Cq) are shown (see Sacchetto et al, 2020, [2]. The immunochromatographic tests (Febre amarela IgM EcoTeste, Dengue IgG/IgM EcoTest, and Zika IgG/IgM EcoTeste-EcoDiagnóstica, Brazil) were performed with undiluted liver homogenate.

The results confirmed the detection of anti-orthoflavivirus antibodies, including anti-YFV antibodies, in the liver homogenate from samples with confirmed infection by YFV. The rapid lateral flow test used here presents high sensitivity (87.6%) and specificity (98%) according to the manufacturer, which attests to a good probability of differentiating true positive samples from negative ones. Thus, the results obtained from the immunochromatographic tests reinforce the results observed for our PRNT assays, which indicate the presence of antibodies against YFV in liver homogenates. In cases where cross-reactivity is reported among closely related viruses, such as with orthoflaviviruses, PRNT or other confirmatory serological tests should be conducted simultaneously to rule out false-positive results. This approach was applied in our study, where YFV-infected NHP carcasses were positive in both PRNT and IgM tests for YFV. In none of IgM-positive samples, no cross-reactivity was observed with IgM against DENV or ZIKV, indicating the presence of YFV-specific IgM in those carcasses. The detection of IgM against orthoflaviviruses is an important indicator of viral circulation among wild animals in a given area. However, two YFV IgM-positive samples (34%) also tested positive for IgG against DENV or ZIKV, indicating the presence of IgG against orthoflaviviruses in those samples. One sample (ID 205 –Table 5) presenting high neutralizing activity (from 86.47% to 48.82 at dilutions 1:20 up to 1:80) but was negative for YFV by PCR and for IgM. The detection of high neutralization activity without presence of IgM and viral RNA could be linked to later times post-infection, and presence of IgG replacing IgM. Unfortunately, we were not able to test the samples for YFV IgG, using the liver homogenate, and this sample did not cross reacted with IgG or IgM against DENV or ZIKV. Nevertheless, the high levels of neutralization observed for this sample indicate that this animal is suspected for YFV or other orthoflavivirus infection. Coupled with epidemiological data, this type of information can be valuable for both passive and active surveillance strategies.

Our findings demonstrate the viability of using liver homogenate from carcasses to investigate antibodies against three different arboviruses using adapted PRNT. Previous studies have demonstrated that experimental infection of different NHP models with different viral strains of YFV, MAYV, and CHIKV led to similar kinetics of viremia and antibodies in the animals, resembling their respective infections in humans. NHPs experimentally infected with YFV showed viremia from 3–7 dpi, peaking around 4 dpi, and antibodies could be detected from 7 dpi through convalescence phase [3,50–52]. Experimental infection of NHP models with MAYV indicated viremia peaking 2 dpi and virus-specific IgM and IgG detected from 5 dpi [53]. In NHPs experimentally infected with CHIKV, viremia peaked from 2–4 dpi, and antibodies were detected one week after infection, with a similar pattern as observed in humans [54]. These studies demonstrate that antibodies can be detected in NHPs concomitant with or soon after peaks in viremia, reinforcing the chances of detecting antibodies in the liver of NHP carcasses collected in the field. Of the NHPs tested here, the concomitant detection of YFV RNA in the liver and YFV-specific antibodies is congruent with the period of intoxication, characterized by seroconversion, no viremia, and virus replication in the liver [3]. The results presented here suggest the potential for serological investigation using liver homogenates for viral infections that can be assessed by PRNT. On the other hand, prior studies have also shown the detection of antibodies in solid tissues from experimentally infected animals or in blood from human cadavers by ELISA [25,26]. Thus, an alternative strategy would be testing liver homogenates by rapid tests or ELISA whenever available, obviating the need for running PRNT, or using the adapted PRNT as a complementary test. This approach offers potential advantages in terms of cost reduction, reduced testing time and less stringent biosafety requirements for screening samples during surveillance programs.

To avoid false positives in the adapted PRNT, careful attention must be paid when preparing liver homogenate to minimize the presence of particulate organic matter and nonspecific neutralization. Samples should be clarified by centrifugation until no visible pellet remains, followed by inactivation, re-clarification, and filtration. The results of the adapted PRNT using liver homogenate should be interpreted considering the appropriate cutoffs per dilution along with the reduction in neutralization according to the dilution. Given the limitations of the adapted PRNT, positive results should be confirmed by additional tests, whenever possible. On the other hand, it is also crucial to note that even in individuals previously exposed to a virus, antibody levels may be low, potentially leading to false negatives. Additionally, factors such as the phase of infection, the preservation status of the carcasses, and the inherent limitations of the experimental assay could result in false negatives, and the possibility of infection should not be dismissed. Similar challenges arise with molecular tests when investigating viral RNA. Consequently, the use of liver homogenate for antibody detection through adapted PRNT or other serological tests is proposed as a complementary strategy to the state-of-the-art assays used in research or surveillance programs.

Here, we detected antibodies against YFV in liver homogenates of NHPs using rapid tests and PRNT. We observed neutralization activity in samples that were previously confirmed or not for YFV infection by RT-qPCR. During the investigation of outbreaks and epizootics, such as those caused by YFV, molecular or serological tests using serum should be conducted whenever possible to assess infection. However, in some instances serum samples are unavailable or biological samples are unsuitable for molecular testing, leading to inconclusive results. Carcasses are convenient samples that can be used for outbreak investigations, and the potential use of liver homogenates in serological tests, as rapid lateral flow tests, adapted PRNT, ELISA or others expands the possibilities for the investigation of outbreaks and epizootics. A positive result in serological tests, such as rapid tests or the proposed adapted PRNT can trigger further investigations in specific areas, prompting the collection of new samples to investigate viral infections in animals or humans. Therefore, we propose this approach as a complementary strategy for investigating epizootics, particularly those caused by YFV.

## Supporting information

S1 Table Median virus neutralization values (%) of YFV, MAYV, CHIKV using liver homogenates from experimentally infected mice in adapted plaque reduction neutralization tests. Legend: YFV: Yellow fever virus, MAYV: Mayaro virus, CHIKV: Chikungunya virus. ID: sample identification. Median virus neutralization values using liver homogenates in adapted plaque reduction neutralization assays are shown in percentages (results from three independent assays: A1-A3). YFV-, MAYV-, and CHIKV- infected groups consisted of six mice (i1-i6) as well as the respective mock-infected groups (m1-m6).
(XLSX)

S2 Table Results of generalized linear mixed models’ analysis of the response of average virus neutralization to virus, dilution and virus/dilution in adapted plaque reduction neutralization tests using liver homogenates of experimentally infected or mock mice. Legend: ^**a**^complete significant model, ^b^complete non-significant model, ^c^minimal adequate model.
(XLSX)

S3 Table Median virus neutralization values (%) of ZIKV per dilution and group, using liver homogenates from experimentally yellow fever virus-infected mice in adapted plaque reduction neutralization tests. Legend: YFV: Yellow fever virus, ZIKV: Zika virus. Median virus neutralization values using liver homogenates in adapted plaque reduction neutralization assays are shown in percentages (results of triplicates). YFV-infected groups consisted of three mice. The negative control is liver homogenate obtained from uninfected animals (Neg), the positive control is sera from experimentally infected animal (pos 1), and the second positive control is composed of a mixture (1:1) of liver homogenate of uninfected animal and positive serum (pos2).
(XLSX)

S4 Table Receiver operating characteristic (ROC) curve analyses for the lowest median neutralization values of YFV-, MAYV, CHIKV-infected groups compared to the highest median neutralization values of respective mock groups. Legend: Lowest (for infected groups) and highest (for mock groups) median neutralization values using liver homogenates in adapted plaque reduction neutralization assays were used for ROC analyses. Samples were grouped (n = 12; 6 infected + 6 mock) and compared by dilution. Analyses were made for each virus separately and considering all data together (n = 36). YFV: Yellow fever virus, MAYV: Mayaro virus, CHIKV: Chikungunya virus. AUC: area under the curve. dil: dilution. Analyses were carried out with Graph PadPrism 8.0.2. The asterisks indicate statistical significance.
(XLSX)

S5 Table Investigation of antibodies against yellow fever virus, and Zika virus using adapted neutralization test, using liver homogenates from non-human primate carcasses. Legend: RT-qPCR: reverse transcription polymerase chain reaction. PRNT: plaque reduction neutralization test. sample identification. P: positive; N: negative and -: not available. YFV: Yellow fever virus. ZIKV: Zika virus. *A*. *guariba*: *Alouatta guariba; C*. *penicillata*: *Callithrix penicillata; C*. *geoffroyi*: *Callithrix geoffroyi; C*. *personatus*: *Callicebus personatus; C*. *nigrifrons*: *Callicebus nigrifrons*. Carcasses were previously classified regarding the preservation status as good (G), intermediate (I), or bad (B) (see Sacchetto et al, 2020, reference 2). Results of RT-qPCR performed on NHP liver/or brain samples with information on the cycle quantification value (Cq) are shown (see Sacchetto et al, 2020, reference 2). The neutralization values observed in adapted PRNT using YFV or ZIKV are show per dilution.
(XLSX)

S1 Fig Cytotoxicity by MTT assay in C57BL/6 and IFNAR^-^/^-^ animals. Legend: Liver homogenates of C57BL/6 and IFNAR^-^/^-^ animals were incubated in Vero cells CCL-81 for two and five days, respectively. Then, the MTT cytotoxicity was evaluated by absorbance reading. For two days of incubation (grey bars), the mean cell viability was equivalent to 80.13% at dilution 1:20 and over 80% in higher dilutions. For five days of incubation (black bars), the mean cell viability was 75.97% at dilution 1:20 and over 80% at higher dilutions.
(TIF)

S2 Fig Comparison of average virus neutralization in adapted plaque reduction neutralization tests using liver homogenates of YFV-, MAYV-, and CHIKV-infected mice. Legend: (A) and (C) Estimates of average viral neutralization observed in relation to dilution of virus, and the type of virus, respectively. (B) and (D). Pairwise analyses indicated differences between all serial dilutions, with the decrease of average neutralization values with increasing dilutions (p< 1.0−6, A and B). Regardless of dilution, MAYV presented higher average neutralization values compared to YFV (p< 0.01) and CHIKV (p = 0.044), while no difference was observed between CHIKV and YFV (p = 0.62, C and D). Results of post-hoc analyses of generalized linear mixed models, with Tukey correction. YFV-, MAYV-, and CHIKV- infected groups consisted of six mice each. YFV: Yellow fever virus (in blue), MAYV: Mayaro virus (in green), CHIKV: Chikungunya virus (in orange). The asterisks indicate statistical significance.
(TIF)

S3 Fig Comparison of average virus neutralization in adapted plaque reduction neutralization tests using liver homogenates obtained from mock-infected mice tested against YFV, MAYV, and CHIKV. (A) Estimates of average viral neutralization observed in mock groups tested against YFV, MAYV and CHIKV at dilutions 1:20 to 1:160. YFV-, MAYV-, and CHIKV- mock groups consisted of six mice each. (B) Results of post-hoc analyses of generalized linear mixed models, with Tukey correction. Post-hoc pairwise comparisons demonstrated that the mock group tested against CHIKV exhibited lower values of nonspecific neutralization compared to the mock groups tested against YFV and MAYV at dilution 1:20 (p< 0.01). Conversely, at dilutions 1:40 to 1:160, mock animals tested against CHIKV and YFV presented similar neutralization values compared to each other (p≥0.97). While YFV and MAYV had similar neutralization values at dilution 1:20, the effect of nonspecific neutralization was higher for MAYV compared to YFV at dilutions 1:40 and 1:80 (p≤ 0.03), and compared to CHIKV at dilutions 1:20 and 1:40 (p≤ 0.01). The asterisks indicate statistical significance, and the bold numbers indicate the comparison between groups in the respective dilution. YFV: Yellow fever virus (in blue), MAYV: Mayaro virus (in green), CHIKV: Chikungunya virus (in orange). The asterisks indicate statistical significance.
(TIF)
