# Supplementary figures and images for: Detection of neutralizing antibodies against arboviruses from liver homogenates
Source: PLoS Negl Trop Dis. 2024 Dec 13;18(12):e0012740. doi: 10.1371/journal.pntd.0012740 (PMC11676865; doi:10.1371/journal.pntd.0012740)

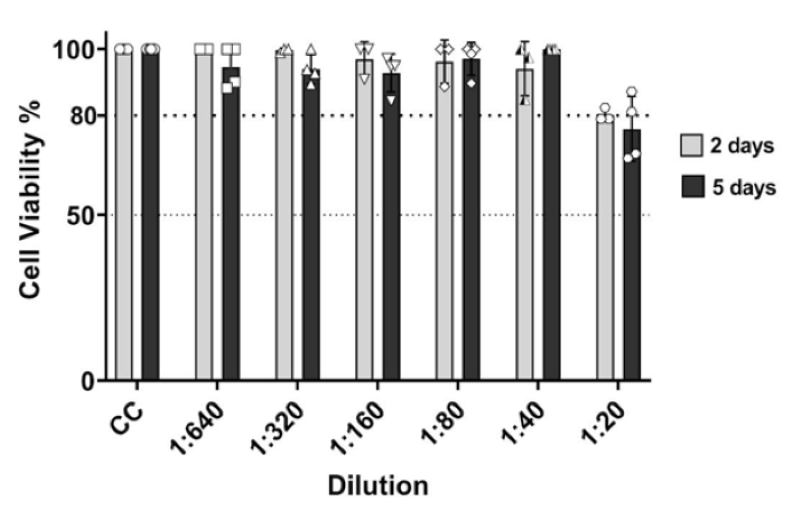

Supplement: S1 Fig — Legend: Liver homogenates of C57BL/6 and IFNAR-/- animals were incubated in Vero cells CCL-81 for two and five days, respectively. Then, the MTT cytotoxicity was evaluated by absorbance reading. For two days of incubation (grey bars), the mean cell viability was equivalent to 80.13% at dilution 1:20 and over 80% in higher dilutions. For five days of incubation (black bars), the mean cell viability was 75.97% at dilution 1:20 and over 80% at higher dilutions. (TIF) [file pntd.0012740.s006.tif]

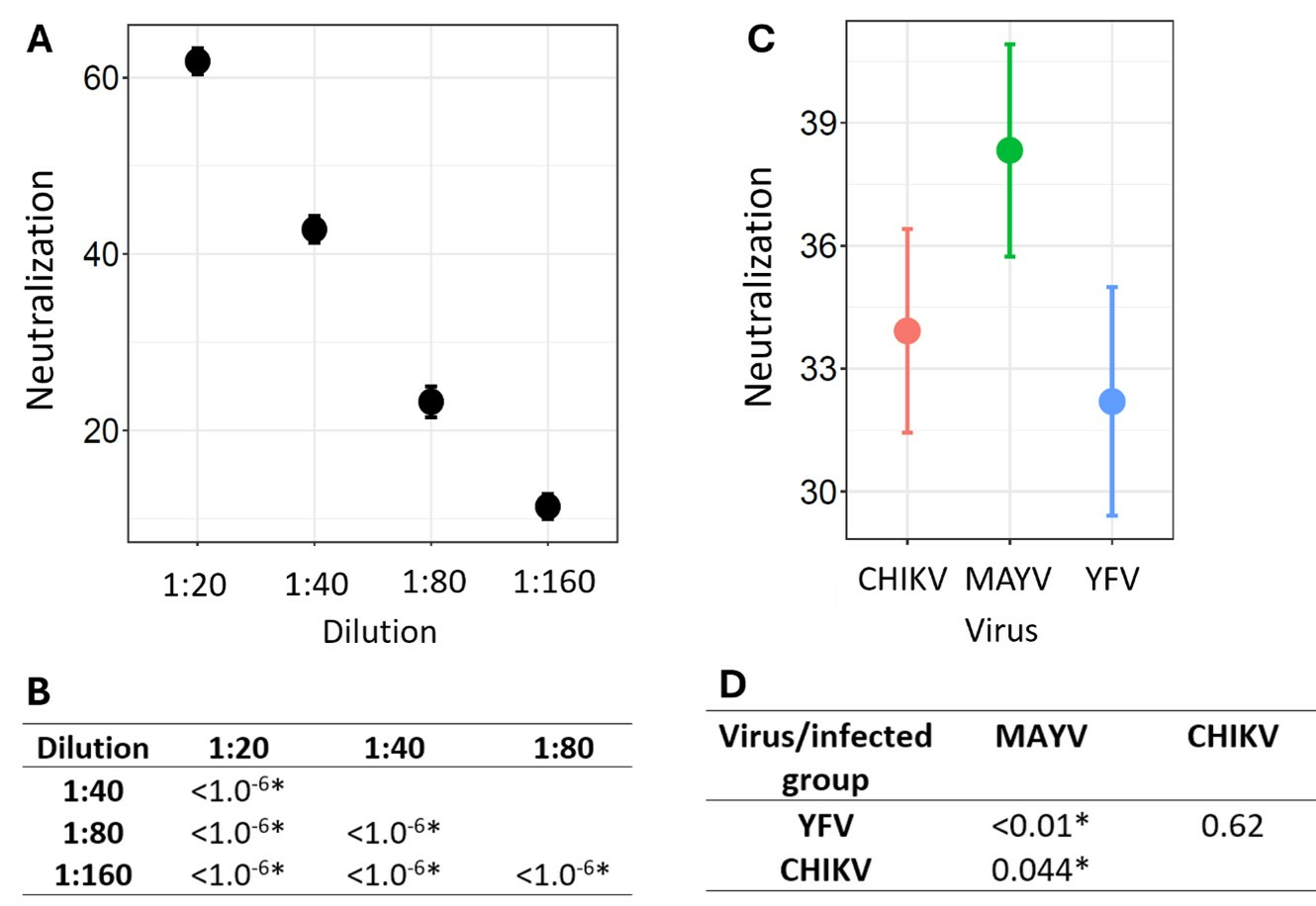

Supplement: S2 Fig — Legend: (A) and (C) Estimates of average viral neutralization observed in relation to dilution of virus, and the type of virus, respectively. (B) and (D). Pairwise analyses indicated differences between all serial dilutions, with the decrease of average neutralization values with increasing dilutions (p< 1.0−6, A and B). Regardless of dilution, MAYV presented higher average neutralization values compared to YFV (p< 0.01) and CHIKV (p = 0.044), while no difference was observed between CHIKV and YFV (p = 0.62, C and D). Results of post-hoc analyses of generalized linear mixed models, with Tukey correction. YFV-, MAYV-, and CHIKV- infected groups consisted of six mice each. YFV: Yellow fever virus (in blue), MAYV: Mayaro virus (in green), CHIKV: Chikungunya virus (in orange). The asterisks indicate statistical significance. (TIF) [file pntd.0012740.s007.tif]

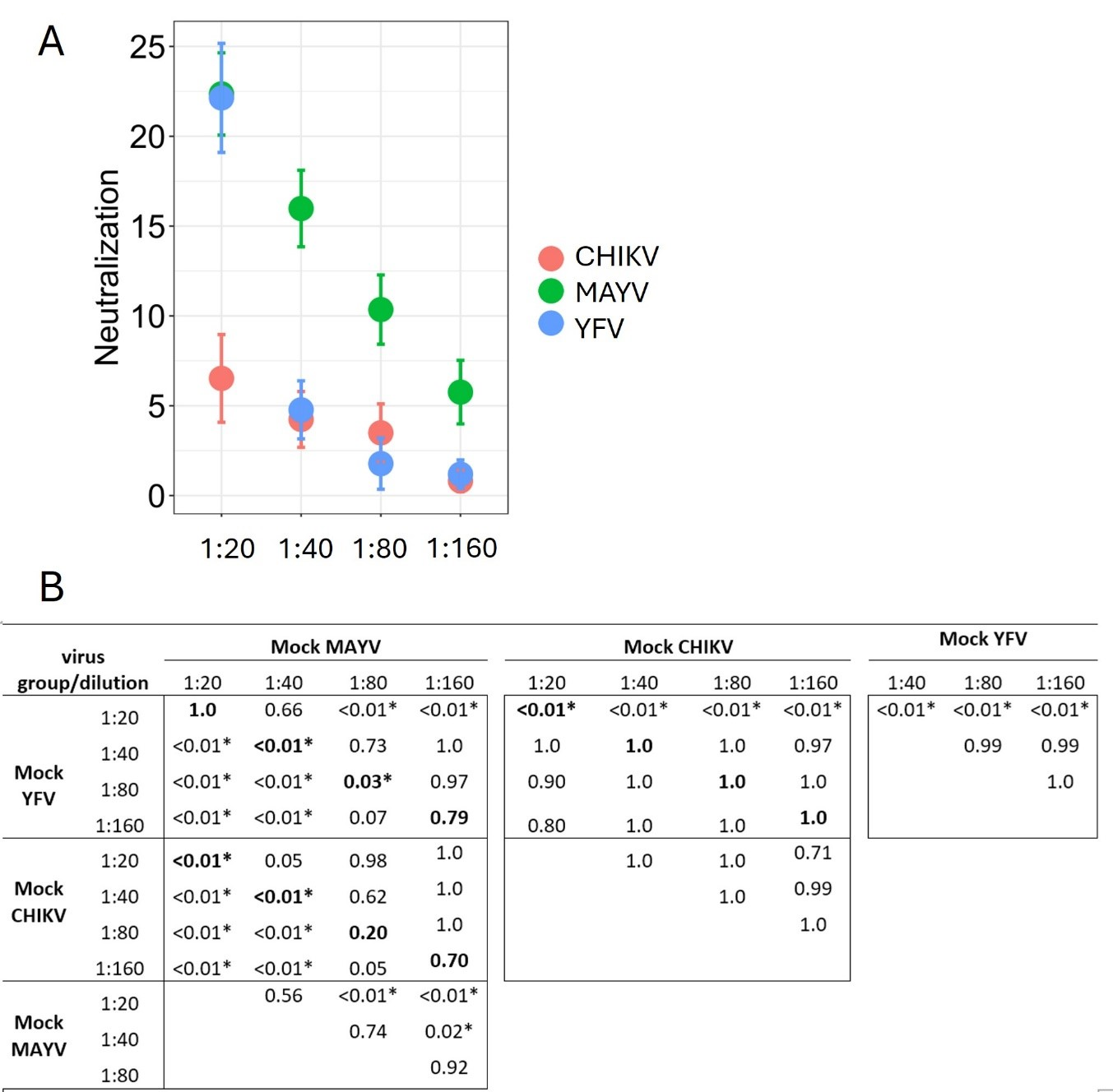

Supplement: S3 Fig — (A) Estimates of average viral neutralization observed in mock groups tested against YFV, MAYV and CHIKV at dilutions 1:20 to 1:160. YFV-, MAYV-, and CHIKV- mock groups consisted of six mice each. (B) Results of post-hoc analyses of generalized linear mixed models, with Tukey correction. Post-hoc pairwise comparisons demonstrated that the mock group tested against CHIKV exhibited lower values of nonspecific neutralization compared to the mock groups tested against YFV and MAYV at dilution 1:20 (p< 0.01). Conversely, at dilutions 1:40 to 1:160, mock animals tested against CHIKV and YFV presented similar neutralization values compared to each other (p≥0.97). While YFV and MAYV had similar neutralization values at dilution 1:20, the effect of nonspecific neutralization was higher for MAYV compared to YFV at dilutions 1:40 and 1:80 (p≤ 0.03), and compared to CHIKV at dilutions 1:20 and 1:40 (p≤ 0.01). The asterisks indicate statistical significance, and the bold numbers indicate the comparison between groups in the respective dilution. YFV: Yellow fever virus (in blue), MAYV: Mayaro virus (in green), CHIKV: Chikungunya virus (in orange). The asterisks indicate statistical significance. (TIF) [file pntd.0012740.s008.tif]
